# Supplementary material for: Evaluation of Selected Medicinal, Timber and Ornamental Legume Species’ Seed Oils as Sources of Bioactive Lipophilic Compounds
Source: Molecules. 2023 May 9;28(10):3994. doi: 10.3390/molecules28103994 (PMC10221283; doi:10.3390/molecules28103994)
Supplement: Supplementary file 1 [file molecules-28-03994-s001.zip › molecules-2313789-supplementary/molecules-2313789-supplementary/Supplementary Figure and Tables.pdf]

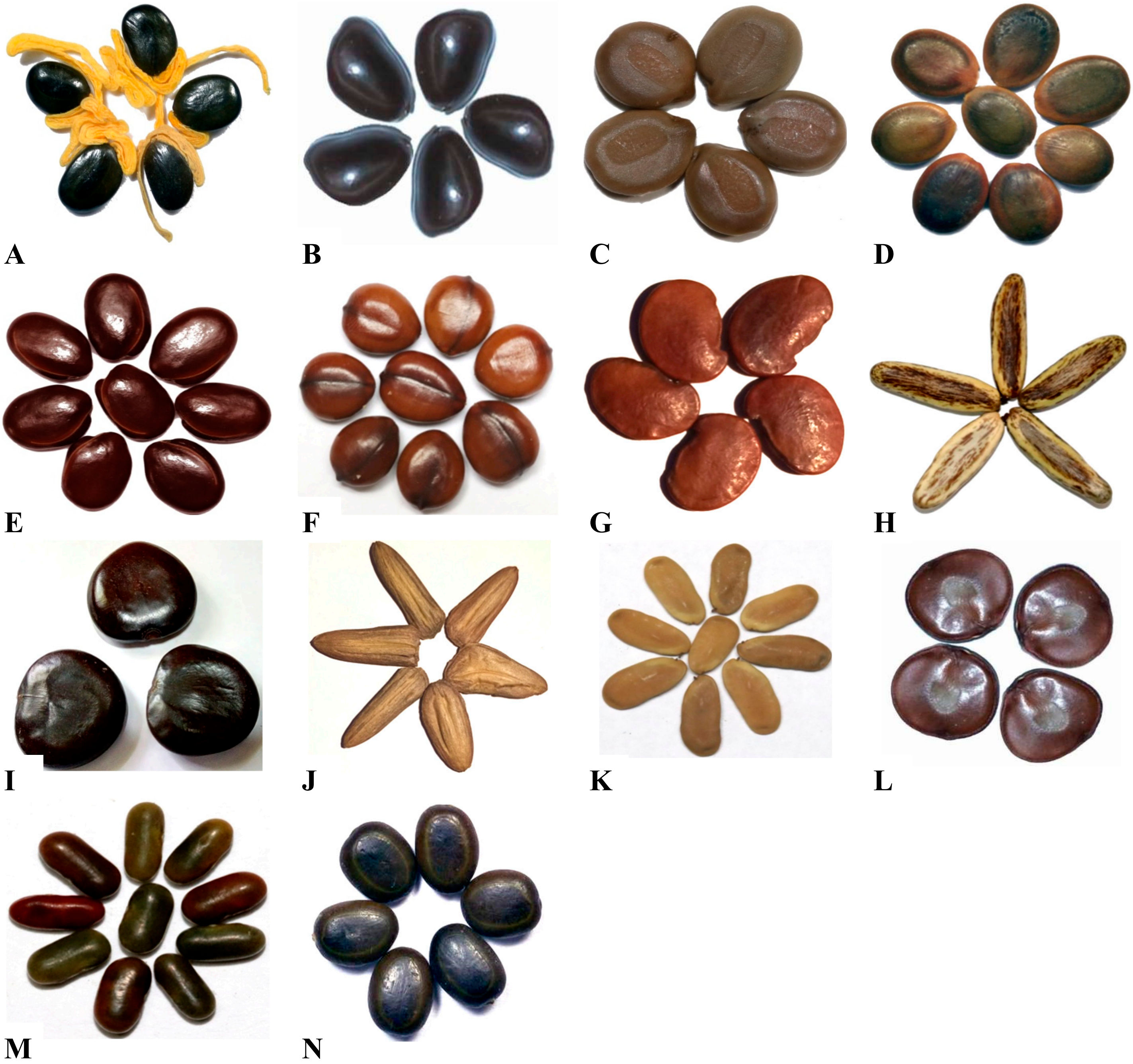

Figure S1. The seeds of fourteen selected tree species belonging to the Fabaceae family: *Acacia auriculiformis* (A), *Acacia concinna* (B), *Albizia lebbeck* (C), *Albizia odoratissima* (D), *Bauhinia racemosa* (E), *Cassia fistula* (F), *Dalbergia latifolia* (G), *Delonix regia* (H), *Entada phaseoloides* (I), *Hardwickia binata* (J), *Peltophorum pterocarpum* (K), *Senegalia catechu* (L), *Sesbania sesban* (M), *Vachellia nilotica* (N).

Table S1. Physical characteristics of the seeds of fourteen selected tree species belonging to the Fabaceae family.

Species	Colour	Shape	Seed mass, mg
A. auriculiformis	Brown	Oblong-ovate	33 ± 2
A. concinna	Brown	Ovate-globular	224 ± 5
A. lebbeck	Brown	Flat-elliptic	74 ± 1
A. odoratissima	Brown	Oblong-elliptic	159 ± 3
B. racemosa	Reddish-brown	Ovoid-compressed	206 ± 5
C. fistula	Light brown	Flattened ovate	292 ± 6
D. latifolia	Light brown	Reniform-flattened	93 ± 3
D. regia	Glossy brown	Oblong	510 ± 5
E. phaseoloides	Dark red-brown	Heart shaped	23623 ± 30
H. binata	Light brown	Oblong ovate	295 ± 5
P. pterocarpum	Light brown	Oblong	94 ± 10
S. catechu	Brown	Elliptic-ovate	57 ± 3
S. sesben	Brownish green	Narrow kidney shaped	15 ± 1
V. nilotica	Brown	Circular-ovate	198 ± 4

Table S2. Sum of SFA, MUFA, PUFA and UFA (%) and fatty acids ratios in the seed oils of fourteen selected tree species belonging to the Fabaceae family.

Species	ΣSFA	ΣMUFA	ΣPUFA	ΣUFA	Fatty acids ratio ΣUFA/ΣSFA
A. auriculiformis	18.9	25.3	55.7	81.1	4.3
A. concinna	11.3	33.0	55.7	88.7	7.8
A. lebbeck	30.0	23.0	47.0	70.0	2.3
A. odoratissima	21.3	14.4	64.3	78.7	3.7
B. racemosa	11.1	34.8	54.1	89.0	8.0
C. fistula	10.4	34.7	54.9	89.6	8.6
D. latifolia	27.0	23.3	49.7	73.0	2.7
D. regia	27.5	14.9	57.6	72.5	2.6
E. phaseoloides	14.9	25.3	59.8	85.1	5.7
H. binata	36.1	21.7	42.3	63.9	1.8
P. pterocarpum	33.1	20.0	47.0	66.9	2.0
S. catechu	24.3	33.1	42.6	75.7	3.1
S. sesben	11.6	33.3	55.1	88.4	7.6
V. nilotica	20.2	26.4	53.4	79.8	4.0

ΣSFA, sum of saturated fatty acids; ΣMUFA, sum of monounsaturated fatty acids; ΣPUFA, sum of polyunsaturated fatty acids; ΣUFA, sum of unsaturated fatty acids.

Table S3. Content of squalene and total carotenoids, sterols, and tocochromanols (mg/100 g dw seeds) in the seeds of fourteen selected tree species belonging to the Fabaceae family.

Species	Squalene	Total		
		Carotenoids	Sterols	Tocochromanols
A. auriculiformis	nd	3.2 ± 0.2	45.2 ± 1.8	14.7 ± 0.6
A. concinna	nd	0.1 ± 0.0	44.5 ± 3.7	2.2 ± 0.2
A. lebbeck	nd	1.5 ± 0.1	33.1 ± 1.4	14.4 ± 0.9
A. odoratissima	nd	1.1 ± 0.1	31.7 ± 1.5	16.5 ± 0.1
B. racemosa	1.1 ± 0.1	0.3 ± 0.0	31.5 ± 1.5	5.8 ± 0.3
C. fistula	9.5 ± 0.3	0.3 ± 0.0	43.8 ± 1.8	7.8 ± 0.3
D. latifolia	nd	0.4 ± 0.0	42.7 ± 1.8	30.8 ± 1.4
D. regia	nd	0.4 ± 0.0	39.0 ± 1.6	11.3 ± 0.1
E. phaseoloides	nd	tr	14.7 ± 0.7	6.1 ± 0.2
H. binata	nd	0.7 ± 0.0	127.9 ± 5.9	43.3 ± 1.7
P. pterocarpum	nd	0.8 ± 0.0	53.6 ± 2.5	16.1 ± 0.4
S. catechu	nd	0.7 ± 0.0	74.9 ± 2.9	7.6 ± 0.2
S. sesben	2.0 ± 0.1	1.1 ± 0.1	47.1 ± 1.7	10.1 ± 0.3
V. nilotica	2.8 ± 0.2	0.2 ± 0.0	47.1 ± 1.6	8.1 ± 0.3

Nd, not detected, tr, trace amount.
